# Ethical-organizational challenges and the repercussions on the health of federal higher education institution employees

**DOI:** 10.47626/1679-4435-2025-1403

**Published:** 2025-07-08

**Authors:** Carolina Souza Nogueira, Jaqueline Gomes de Jesus

**Affiliations:** 1 Graduate Program in Bioethics, Applied Ethics, and Public Health, Escola Nacional de Saúde Pública Sérgio Arouca, Fundação Oswaldo Cruz, Rio de Janeiro, RJ, Brazil

**Keywords:** organizational ethics, occupational health, occupational diseases., ética organizacional, saúde ocupacional, doença ocupacional.

## Abstract

Federal institutions of higher education play an essential role in academic training, research production, and the development of outreach activities, requiring intense interaction between professors, students, and administrative staff. However, these environments face ethical-organizational challenges that significantly affect the physical and mental health of employees. To this end, this review analyzed the literature on ethical-organizational challenges faced by these institutions and their relationship with employee health and illness. A systematic review of studies published between 2010 and 2024 was conducted, including studies that specifically addressed the relationship between ethical-organizational challenges and health in the context of federal institutions of higher education. The main challenges included work overload, pressure for academic productivity, moral harassment, lack of institutional support, and lack of transparency in management. These conditions increase stress, burnout, and mental disorders among employees. However, organizational practices that promote ethics, equity, and professional appreciation contribute to greater engagement and better occupational health conditions. The findings highlight the need to strengthen organizational ethics, train managers, implement effective codes of conduct, and adopt inclusive institutional policies, suggesting that more equitable and sustainable work environments can minimize negative effects on worker health.

## Abstract (pt)

As Instituições Federais de Ensino Superior desempenham papel fundamental na formação acadêmica, na produção científica e no desenvolvimento de atividades de extensão, o que demanda intensa interação entre docentes, discentes e servidores administrativos. No entanto, esses ambientes enfrentam desafios ético-organizacionais que impactam significativamente a saúde física e mental dos servidores. Diante disso, este artigo buscou analisar a produção científica relacionada a tais desafios nessas instituições e sua relação com a saúde e o adoecimento dos servidores. Realizou-se uma revisão sistemática de estudos publicados entre 2010 e 2024, incluindo pesquisas que abordam especificamente a relação entre desafios ético-organizacionais e saúde no contexto das instituições federais de ensino superior. Os principais desafios identificados foram: sobrecarga de trabalho, pressão por produtividade acadêmica, assédio moral, falta de suporte institucional e ausência de transparência na gestão. Tais condições elevam os índices de estresse, *burnout* e transtornos mentais entre os servidores. Por outro lado, práticas organizacionais pautadas na ética, na equidade e na valorização profissional contribuem para o aumento do engajamento e para a melhoria das condições de saúde ocupacional. Os achados evidenciam a necessidade de fortalecer a ética organizacional, capacitar gestores, implementar códigos de conduta eficazes e adotar políticas institucionais inclusivas. O estudo indica que ambientes laborais mais equitativos e sustentáveis podem atenuar os impactos negativos sobre a saúde dos trabalhadores.

## INTRODUCTION

Federal institutions of higher education are fundamental spaces for academic training, the production of scientific research, and the development of extension activities, which requires intense interaction among faculty, students, and administrative staff.^1^ However, these environments face ethical and organizational challenges that significantly affect the physical and psychological health of their employees. Problems such as work overload, inadequate management practices, moral harassment, pressure for academic and financial results, as well as an organizational culture focused on productivity to the detriment of well-being,^2,3^ have contributed to increased occupational and psychosocial illness.

As defined by the World Health Organization, health transcends the mere absence of disease, being understood as a state of complete physical, mental, and social well-being.^4^ This concept is reinforced by the Brazilian Federal Constitution, which recognizes health as a right for all that must be guaranteed universally and equitably through social and economic policies that promote health protection, reduce the risks of illness, and ensure dignified conditions for recovery in cases of illness.^5^ The constitution also guarantees work as a fundamental right, in that it is related to human dignity, freedom, and equality of conditions, guaranteeing protection against unethical and disrespectful practices in the workplace.^5,6^

The *Código de Ética Profissional do Servidor Público Civil* (Code of Professional Ethics for Civil Servants) reinforces the relationship between health and work by emphasizing that civil servants must look after their own well-being and act ethically both in their professional conduct and in the private sphere, contributing positively to the institutional environment. Thus, quality of life at work is understood as a key element in promoting the health of civil servants, especially considering that work is one of the social determinants of health, alongside factors such as diet, housing, income, education, sanitation, and access to essential services.^7-9^

However, modern organizations face a fundamental paradox: on the one hand, they need to be efficient, productive, and competitive in a globalized market; on the other, they are increasingly required to adopt ethical behavior, transparency, and social responsibility. According to Enriquez,^2^ this dilemma generates a series of ethical challenges, such as the instrumentalization of ethics as a tool, i.e., when companies employ ethical discourse only to maintain legitimacy, without promoting structural change; instrumental rationality, which leads to the alienation of workers, reducing them to mere productive resources and disregarding their subjectivity; organizational hypocrisy, which manifests itself when companies preach values such as diversity and well-being but maintain contradictory practices; and the influence of capitalism, which imposes superficial ethics based on standards and certifications, without a real commitment to moral values.

Thus, the dynamics of inequality and organizational practices that contribute to illness can also be found in federal institutions of higher education. This situation can be aggravated by a lack of adequate institutional support and a lack of prioritizing ethics and worker health by management. Problems such as precarious working conditions, work overload, and a lack of inclusive policies aggravate the negative impacts on the physical and mental health of employees.^3^

Work, although a central element in the organization of social life, can also be a health risk factor, especially in work contexts marked by structural inequalities. In this scenario, ethical-organizational practices become essential to mitigate negative impacts and promote healthy work environments. Organizational ethics, based on shared values and the promotion of collective well-being, must go beyond rigid rules and include dialogue, respect for differences, and the search for humane solutions, balancing institutional and individual interests.^2,3^

This article analyzed scientific production related to the ethical-organizational challenges faced by federal institutions of higher education and their relationship with employee health and illness. To this end, a systematic review of the literature published between 2010 and 2024 was performed to identify recurring patterns in these contexts and propose practical solutions based on scientific evidence. The analysis considers the interaction between ethical, organizational factors, and social determinants of health, highlighting how inadequate work practices can compromise not only individual well-being, but also the efficiency and sustainability of institutions.

This review can also contribute to strategies for positive change in the work environments of federal institutions of higher education. By prioritizing ethical, humane, and inclusive organizational practices, institutions can mitigate inequalities, reduce rates of occupational illness, and strengthen worker quality of life. Coordinated and evidence-based actions can promote an organizational culture that values dialogue, equity, and occupational health,^2,3^ ensuring that federal institutions of higher education fulfill their social role in an ethical and sustainable manner.

## METHODS

This study is an integrative literature review, a method recognized for its ability to synthesize results of scientific studies on a specific topic, contributing to a comprehensive and in-depth understanding of the investigated problem.^10^ The integrative design is one of the most comprehensive methodologies for reviews, as it allows the inclusion of different types of studies, facilitating the identification of gaps in knowledge and the proposition of evidence-based solutions.

This type of review not only systematizes existing knowledge but also provides a solid basis for researchers and other interested parties to explore the topic, expand discussion, and seek answers to relevant questions. To achieve its objectives, the method follows six interconnected steps: defining the guiding question, which guides the entire research process; identifying and carefully selecting studies from the databases; extracting and organizing the collected data; critically assessing the included studies, ensuring the quality of the findings; interpreting and discussing the results; and synthesizing and presenting the review, providing a consolidated view on the topic.^10^ More than identifying challenges, integrative reviews seek to propose well-founded solutions that encourage the implementation of inclusive and humane organizational practices. This methodological approach allows the analysis of a topic from multiple perspectives, promoting an integrated view of the involved ethical and social aspects. By identifying paths for health promotion in the workplace, this study reinforces the importance of ethical management, focused on the collective well-being and appreciation of workers.

This study’s guiding question was to understand how ethical-organizational challenges in federal institutions of higher education influence employee health and illness. To answer this question, a systematic search was conducted for articles published between 2010 and 2024 in Portuguese, English, or Spanish. Studies specifically addressing the relationship between ethical-organizational challenges and health in the context of federal institutions of higher education were included. The exclusion criterion was studies that addressed contexts outside public higher education institutions or that did not present a clear and direct connection between ethical-organizational challenges and health. This was to ensure that the analysis was focused and relevant, contemplating only studies that directly responded to the study question.

The search was conducted in the Scopus, Web of Science, Google Scholar, SciELO and CAPES databases, using descriptors such as: “ethical-organizational challenges”; “health at work”; “health of civil servants”; “Federal Higher Education Institutions”; “psychosocial illness” and “management in public universities”; “ethical management”; “illness at work” and “health of civil servants”; “ethical-organizational risks”; “ethical leadership”; “ethical-organizational culture”; “challenges of managers in federal institutions of higher education.”

Despite defining rigorous criteria in the systematic search process, no articles were found that directly addressed the relationship between ethical-organizational challenges and health in the context of federal institutions of higher education according to the established parameters. This highlights a gap in the literature and reinforces the need for future research to further investigate this interrelationship, given its relevance to occupational health and organizational ethics. Nevertheless, we maintained the study proposal, considering and emphasizing its relevance and the need for in-depth research in the area. We followed an integrative review selection process, searching for articles that demonstrated the relationship between organizational issues in the work environment and occupational illness processes among federal employees. Our objective was to characterize, highlight, and classify the reported ethical challenges, if any.

After this stage, articles not published in indexed journals were excluded, as were review articles, theses, dissertations, course completion papers, book chapters and other documents that did not adequately fit the objective of this review. It was also decided to exclude articles prior to 2020 to select only those prepared in the context of work during and after the pandemic period. The article selection process was conducted in three stages: title and abstract reading, full text reading of the selected articles, and qualitative analysis to categorize the evidence. Preferred Reporting Items for Systematic Reviews and Meta-Analyses guidelines were followed, ensuring the rigor and transparency of the process.

After study selection, the analysis prioritized investigations that addressed, even indirectly, the relationship between organizational ethics and occupational health in determining the quality of life and working conditions of workers. Issues such as overwork, inadequate management practices, moral harassment, structural inequalities, and lack of institutional support were considered. The critical analysis highlighted the impact of these conditions on the mental and physical health of employees, showing how organizational ethics can be a central element in the transformation of work environments. At this point, six studies that addressed the topic more specifically were selected and carefully analyzed, contributing to a more objective answer to the guiding question. Chart 1 shows the author(s), year of publication, title, methods, objectives, and conclusions, on which the review’s discussion and conclusion were based.

**Chart 1:** Articles selected for integrative review

Authors	Title	Methods	Objectives	Conclusions
Costa^11^	A influência do contexto de trabalho na saúde de servidores de instituição federal de ensino superior	Exploratory study with documentary analysis, application of the Inventário de Trabalho e Riscos de Adoecimento and semi-structured interviews.	To analyze the influence of the work context on the health of employees of federal higher education institutions.	The work context affects the physical, mental and social health of employees, requiring both individual and organizational alternatives.
Tessarini Junior et al.^12^	Avaliação do contexto de trabalho em uma instituição federal de ensino	Case study with documentary analysis, participant observation, and application of the Escala de Avaliação do Contexto de Trabalho.	To analyze the work context of technical-administrative staff in the area of ​​people management.	The work context is critical, producing suffering and the need for defense strategies to minimize health impacts.
Souza et al.^13^	Trabalho docente, desigualdades de gênero e saúde em universidade pública	Qualitative research from the perspective of materialist feminism with thematic content analysis.	To problematize teaching work in relation to gender, assessment policies, and health.	The sexual division of labor and assessment policies lead to overload and compromise health, especially for women.
Tessarini Junior & Saltorato^14^	Organização do trabalho dos servidores técnico-administrativos em uma instituição federal de ensino: uma abordagem sobre carreira, tarefas e relações interpessoais	Case study with 18 semi-structured interviews, analyzed using a qualitative approach.	To explore the experiences of TAS and their perceptions about career, tasks, and interpersonal relationships.	Conflicts between professional expectations and working conditions compromise the well-being of TAS.
Silva & Silva-Costa^15^	Presenteeism and psychosocial aspects of work among civil servants in leadership positions at a Brazilian federal university	Cross-sectional study with 106 managers using an online questionnaire and binomial logistic regression.	To assess the relationship between stress, burnout syndrome, and presenteeism among managers.	Presenteeism, which is high among managers, is associated with low productivity and chronic stress.
Sobreira et al.^16^	Comprometimento organizacional: estudo com servidores técnico-administrativos de nível superior da Universidade Federal de Viçosa	Quantitative descriptive study that applied a questionnaire to 195 employees and performed statistical analysis.	To describe and analyze organizational commitment among TAS.	Organizational commitment is high, with a greater emotional bond between more experienced employees.

TAS = technical-administrative staff.

## RESULTS

Ethical-organizational challenges in federal institutions of higher education have been recognized as a critical factor in the health and well-being of employees. Comparative analysis of the selected articles showed that management practices, institutional policies, and the organizational context are central elements in understanding this impact.

The studies emphasize the influence of the work context on health, especially regarding work overload, pressure for results, and precarious working conditions, which negatively affect the physical and mental health of workers. Although Costa’s article^11^ did not directly address ethical issues, it suggested that working conditions may be worsened by the absence of organizational practices that prioritize the dignity and well-being of employees, highlighting the need for more solid organizational ethics to face these challenges.

Such organizational and management practices, as well as their relationship with occupational suffering, were analyzed by Tessarini Junior et al.^12^ in the work experience of technical-administrative staff. High levels of suffering were identified due to ineffective management practices and lack of institutional support. The authors addressed ethical issues by criticizing the lack of organizational strategies to humanize the work environment. They pointed out the lack of transparency and equity in management decisions as one of the main sources of ethical conflict, worsening the mental health impact on employees.^12^

With a critical perspective on the sexual division of labor in federal institutions of higher education, Souza et al.^13^ analyzed how institutional policies reinforce gender inequalities and compromise the mental health of female teaching staff, observing how these inequalities are not only structural, but ethical as well, since they perpetuate discriminatory and dehumanizing practices. The absence of inclusive policies that promote equity reflects a deficit in organizational ethics that directly affects occupational health.

Regarding ethical conflicts, Tessarini Junior & Saltorato^14^ explored interpersonal relationships and the challenges faced by administrative staff in federal institutions of higher education, pointing out that interpersonal conflicts, which are often due to a lack of communication and inadequate management, are manifestations of organizational ethical problems. Competition between peers, combined with a lack of ethical leadership that promotes cooperation and respect, is detrimental to the work environment and compromises employee health.

Supporting this perspective, da Silva & Silva-Costa^15^ analyzed presenteeism and psychosocial aspects of work among managers of federal institutions of higher education, indicating that occupational stress and burnout syndrome are associated with high work demands and low social support. This study also reported that the lack of ethical management practices, particularly those related to safeguarding worker health and balancing organizational demands with individual capabilities, contributes to occupational illness and psychosocial harm. Ethics in leadership is a fundamental element in minimizing negative effects in the work environment.

Sobreira et al.^16^ investigated the organizational commitment of technical-administrative staff, finding that an emotional bond with the institution is related to better mental health indicators, which suggests that strengthening organizational ethical values - such as transparency, respect and recognition - is essential to foster employee commitment and promote a healthier environment. A lack of alignment between institutional values and organizational practices can result in dissatisfaction and intensify ethical conflicts.

The studies converged on the finding that ethical-organizational challenges in federal institutions of higher education significantly affect the physical and mental health of employees. Although some articles only indirectly addressed ethical issues, the lack of humanized organizational practices was a factor observed throughout the studies. The literature indicates that shortcomings in ethical management practices contribute to work overload, structural inequalities, and strained interpersonal relationships.

More broadly, the findings reinforce the need for an ethical commitment by leaders and institutions that is aligned with the principles of equity, transparency, and respect for diversity. Some studies^2,3^ also corroborated the idea that organizational ethics must go beyond norms and regulations, promoting an environment that prioritizes both collective and individual well-being.

Federal institutions of higher education should invest in ethical leadership that values transparency and dialogue. Training programs focused on ethics at work, strengthening institutional support, and implementing inclusive policies are promising paths forward. In addition, institutions should conduct ongoing assessments of the impact of their organizational practices on employee health, implementing corrective measures whenever necessary.

## DISCUSSION

Ethical and organizational challenges in federal institutions of higher education are a multifaceted problem, with relevant implications for the health and well-being of their employees. The included studies pointed out issues related to human resource management, interpersonal conflicts, organizational culture, and pressure for productivity, indicating a scenario that demands integrated initiatives based on ethical principles.

The main challenges identified in these studies include: (i) human resource management; (ii) bullying and interpersonal conflicts; and (iii) organizational culture. Work overload, a lack of resources, and a lack of professional recognition were found to directly impact employee motivation and well-being, since such issues reflect flaws in institutional policies and lead to dissatisfaction in the work environment.^6^ It is important to highlight that a lack of effective support from Occupational Health Reference Centers has helped worsen this situation.^17^ Moral harassment, defined by Guia Lilás^18^ as a set of abusive behaviors that expose workers to humiliating situations, compromises the mental and emotional health of workers. It is worth noting that organizational environments marked by systematic episodes of harassment are associated with emotional exhaustion, low self-esteem, and increased absenteeism.^19^ Furthermore, the pressure for academic and financial results creates work environments marked by tension and stress, compromising quality of life at work.^13^ Furthermore, some studies^2,3^ suggest that well-established organizational ethics can act as a mechanism to mitigate these challenges, promoting greater balance between institutional demands and worker needs.

The widely documented effects of ethical-organizational challenges on employee health included: (i) stress and anxiety - a high incidence of stress among employees subjected to excessive demands and a lack of institutional support,^20^ and the lack of organizational support intensifies these situations^18^; (ii) burnout and depression - emotional exhaustion resulting from chronic workplace stress is directly associated with burnout syndrome, indicating that depersonalization and the feeling of professional inefficacy are frequent consequences in hostile work environments^19^; and (iii) physical illnesses - occupational illnesses, such as repetitive strain injuries/work-related musculoskeletal disorders and cardiovascular diseases, are related to adverse working conditions, highlighting the impact of physical and mental work on overall employee health.^21^

The relationship between ethical-organizational challenges and employee illness is widely discussed in the literature, albeit indirectly, suggesting, for example, that organizational structures based on discriminatory and centralizing practices perpetuate historical inequalities, contributing to environments that intensify the negative effects of work on the physical and mental health of workers.^22^ The lack of clear communication and policies focused on well-being reinforces the cycle of illness, making it difficult to build healthy and ethical environments.^2^ The included studies highlight the main factors that contribute to occupational illness in federal institutions of higher education, including: work overload and constant pressure for productivity; authoritarian and centralized management, which inhibits the autonomy and participation of employees; and work environments characterized by a lack of institutional support and conflicting interpersonal relationships.

It is also worth highlighting some of the strategies suggested by the authors to face ethical-organizational challenges and promote the health of employees: i) training in ethical management by implementing training programs that emphasize respectful coexistence, conflict mediation, and strengthening dialogue between employees and managers^3^; ii) well-being policies that establish programs focused on mental health and psychosocial support to promote a healthier and more inclusive work environment^17^; iii) professional appreciation that includes recognizing and rewarding employees for results, streamlining processes, and promoting spaces for active listening. Such practices can contribute to increased motivation and organizational engagement.^2,6^

## CONCLUSIONS

This systematic review demonstrated a direct relationship between ethical-organizational challenges in federal institutions of higher education and negative effects on employee health. Preserving the psychosocial health of workers requires coordinated actions that involve not only public policies, but also ethical management that is sensitive to both collective and individual needs.^2,3,6^

Trained managers and healthy work environments are essential for minimizing the negative effects of work on health. Analysis of organizational structures indicates that inclusive environments, guided by ethics and the promotion of well-being, can lead to significant positive change. As pointed out in previous studies,^3,23^ the creation of safe spaces and the implementation of actions based on ethical values strengthen not only occupational health, but worker engagement as well, promoting greater institutional efficiency.

The results show that ethical and organizational challenges in federal institutions of higher education significantly affect the physical and mental health of employees, indicating the need for an integrated approach that considers not only institutional productivity, but also worker well-being and dignity. The implementation of ethical practices, well-being policies, and initiatives aimed at professional development is essential for institutions to truly incorporate moral values into their practices. This requires recognizing the complexity and limits of human actions, thus promoting a more humane and responsible environment.^2^

The consolidated results can contribute to interventions and policies that aim to create healthier and fairer work environments. This review not only broadens our understanding of ethical and organizational challenges in federal institutions of higher education but also supports improved quality of life for employees and promotes an institutional culture based on ethics and health care.

Therefore, it is essential that federal institutions of higher education adopt concrete measures, such as continuous training for managers, strengthening organizational ethical codes, and developing inclusivity initiatives that respect diversity and individual differences. This study also highlights the relevance of ethical practices as a basis for effective interventions, not only in federal institutions of higher education, but in public and private organizations as well.
